# Morgan County Medical Society

**Published:** 1866-08

**Authors:** R. E. McVey, G. R. Bibb

**Affiliations:** Pres’t.; Jacksonville, Ill.; Sec’y.; Jacksonville, Ill.


### MORGAN COUNTY MEDICAL SOCIETY.

Jacksonville, Ill., May 17, 1866.

The Morgan County Medical Society met at the Court House at 2 o'clock P. M., pursuant to adjournment. The President being in the chair, the minutes of the previous meeting were read and approved.

The President announced the report of the committee on the Constitution to be in order, which was accordingly read by the Secretary.

Dr. Askew nominated Dr. McVey, of Waverley, for President the ensuing year, which being put to the house by the Secretary, was carried by unanimous vote.

On motion of Dr. Stephenson, Dr. J. P. Johnson, of Lynnville, was elected Vice-President.

On motion of Dr. Edgar, Dr. Bibb was chosen Secretary by a unanimous vote.

Dr. J. Craig, of Arcadia, having been nominated for Treasurer, was elected by unanimous vote.

The following gentlemen were elected as Examining Committee: Drs. W. S. Edgar, D. Prince, Henry Jones, C. Fisher and J. Askew.

The President announced the next order of business to be the reading of an essay by Dr. Henry Jones, on Asiatic Cholera. The Doctor having seated himself at the desk, commenced to read his manuscript at the forty-ninth page, (fearing that there would not be time to read all.) He was listened to with marked attention and interest to the conclusion, nearly two hours.

On motion of Dr. Prince, it was resolved that Dr. Jones be solicited to furnish a copy of his dissertation to some medical journal for publication. The thanks of the society were tendered to Dr. Jones for his very able and instructive essay.

On motion of Dr. Edgar, it was resolved to make Asiatic Cholera the subject of general debate at the next meeting of the society. Dr. Prince suggested that Dr. Edgar open that discussion.

On motion, it was resolved that the next meeting of the society should take place at 10 o'clock A. M., in this place, on the second Tuesday in June, to which time the society adjourned.

R. E. McVey, M. D., *Pres't.*
G. R. Bibb, M. D., *Sec'y.*

